# Reply to: Sunlight exposure cannot explain "grue" languages

**DOI:** 10.1038/s41598-023-28281-0

**Published:** 2023-02-01

**Authors:** Mathilde Josserand, Emma Meeussen, Dan Dediu, Asifa Majid

**Affiliations:** 1 Laboratoire Dynamique du Langage, Université Lyon 2-CNRS UMR 5596, Lyon, France; 2 Nijmegen, The Netherlands; 3 Department of Catalan Philology and General Linguistics, University of Barcelona, Barcelona, Spain; 4 University of Barcelona Institute for Complex Systems (UBICS), Barcelona, Spain; 5 Catalan Institute for Research and Advanced Studies (ICREA), Barcelona, Spain; 6 Department of Experimental Psychology, University of Oxford, Oxford, UK

**Keywords:** Human behaviour, Evolution of language

**replying to**: J. Hardy; *Scientific Reports* 10.1038/s41598-023-28280-1 (2023).

## Introduction

Hardy et al.^1^ raise two critical points regarding our original research paper^2^. First, they highlight their important earlier work^3^—which we did not discuss in our original article—that suggests lens brunescence cannot explain color naming due to color constancy. Second, they stress a more “parsimonious” account of color naming that relies on the communicative needs of different cultures. We take this opportunity to discuss their findings in light of other recent studies, and to discuss the potential role of lens brunescence as one factor among many that shapes color naming.

Hardy et al.^1^ propose perceptual mechanisms compensate for the deterioration of color perception due to lens brunescence because old and young people tested in the United States name colors similarly^3^. In their earlier study, they determined the ocular media of 20 participants (10 young and 10 old) and also simulated aging and youth by manipulating a computer screen so that it re-created the retinal stimulation of an observer of the other age group. They found simulating aging changed color naming behavior (as also found earlier in^4^), but actual aging did not^3^. They therefore conclude lens brunescence due to UV-B exposure is not a plausible causal mechanism to affect color naming and the presence or absence of blue in a language.

Since then Walter^5^ used yellow-orange ocular filters to simulate aging equivalent to 20–33 years in a high UV-B incidence environment or 105–165 years European UV-B incidence. Ocular filters, unlike changes to a computer monitor, have the advantage of causing chromatic change for the entire visible environment, akin to what may occur with lens brunescence. Walter found the strongest filters affected naming of blue color patches^5^ which leaves open the possibility that even if there is a compensation mechanism it does not fully offset age-related changes for people exposed to higher levels of UV. Data from NASA’s Total Ozone Mapping Spectrometer for 1998 shows at low latitudes the mean UV incidence is five times higher than central Europe (see also^4^), so the color vision of a 30-year-old adult living in a high UV region corresponds to a putative 150-year-old European. Differences in lifestyle—such as amount of time spent indoors, use of sunglasses and corrective lenses with UV filters by people in higher latitudes—would further exacerbate differences between groups.

It remains plausible, then, that lens brunescence in some populations through its effect on blue color perception could affect color naming. Other studies support this proposal. Color vision deficiencies in the blue range become more common as people age^6–8^, particularly for people living in high UV incidence regions^9,10^. For example, Davies et al.^10^ studied color perception in 597 participants from Africa (Botswana, Malawi, and South Africa) and Europe (Greece, Spain, Ireland, and UK), and found African participants, as well as Greeks to some extent, made more errors in the blue range. The pattern of errors made by African participants resembled those of older British participants, again suggesting lens brunescence could be a causal mechanism.

Moreover, recent agent-based computational models using dynamic systems approaches show in realistically heterogeneous populations of perceivers (i.e., where not everyone shares the identical perceptual apparatus), the presence of dichromats affects color boundaries for the whole population. With as few as 1% dichromats in a population there are changes to color categories for the entire population^11,12^. So, contrary to Hardy et al.^1^, we do not believe the current data on chromatic adaptation rules out the lens brunescence hypothesis. In addition, there is a plausible account for how perceptual heterogeneity can affect color categories within a population. We conclude additional data from participants of different ages at different latitudes is necessary to adjudicate the matter. Like Hardy et al.^1^ we believe “Further empirical research into the rate of brunescence in geographic areas in question would aid our understanding” because the matter is not yet settled.

As an alternative, Hardy et al.^1^ suggest different communicative needs across cultures are sufficient to account for color naming strategies globally. They appeal to the seminal work of Berlin and Kay^13^ which suggests languages develop color categories in a predictable sequence. They go on to suggest technological complexity alone (developed within a culture or acquired through contact) could account for color categories. While this parsimonious account is attractive, we believe it to be insufficient. The data and analyses in our paper suggest two environmental factors—UV-B incidence and distance to lakes—play a role in shaping color lexicons separately from the role of culture. Similarly, Twomey et al.^14^ showed geographic location and ecological region influence color naming. In addition, a recent study of the hunter-gatherer Maniq (Thailand) showed that color lexicons can emerge in response to environmental not technological demands^15^. We agree communicative needs shape color lexicons, but current accounts underspecify the cultural and environmental demands that themselves drive differential communicative needs. This matter deserves further empirical attention.

Hardy et al. claim that since level of technology, distance from the equator (a proxy for low UV-B exposure), and number of basic color terms are correlated, we can discard the correlation between the latter two. However, our statistical analyses using mediation and path analyses show you cannot rule out the additional role UV-B exposure plays in color language. Instead, the analyses support the alternative hypothesis that the effect of UV-B incidence on color language is not mediated by, or the result of, a spurious relationship with any other variables we considered in our original paper (see Supplementary Materials^2^ for more information). In our view color naming is affected by a range of variables. Parsimony while attractive does not align well with the complexity of the problem. A parsimony account applied to our model would mean keeping the lowest number of factors that explain the most variance in the outcome—dropping UV-B incidence makes the explanation worse. Our analyses suggest color language is affected by a range of factors that in turn shape both color perception and communicative need.

In conclusion, while compensatory mechanisms—such as color constancy—exist in color perception, it is possible they only partially offset the effects of aging. There is evidence that people exposed to very high levels of UV radiation develop color vision deficiencies in the blue range. So in our view lens brunescence is still a candidate mechanism for shaping color lexicons. Nevertheless, we agree with Hardy et al. that lens brunescence *alone* is unlikely to explain these cross-linguistic patterns, and that other factors, such as cultural dyeing technologies and historical processes, are also important to take into consideration. This is in line with the main conclusion of our original paper: the color lexicon is the result of many factors—cultural and environmental—that interact with each other in complex ways.
